# Subcutaneous Cyst Due to a Novel Fungus, Aquastroma magniostiolata: A Case Report

**DOI:** 10.7759/cureus.27760

**Published:** 2022-08-07

**Authors:** Archana Y Keche, Radhakrishna Ramchandani, Rakesh Gupta, Anuniti Mathias

**Affiliations:** 1 Microbiology, All India Institute of Medical Sciences, Raipur, Raipur, IND; 2 General Surgery, All India Institute of Medical Sciences, Raipur, Raipur, IND; 3 Pathology, All India Institute of Medical Sciences, Raipur, Raipur, IND

**Keywords:** newer fungal pathogens, subcutaneous cyst, pheohyphomycosis, aquastroma, rare fungi

## Abstract

Various kinds of fungal agents have been observed in the environment. Fungi can enter the human body by penetration following trauma and are responsible for various types of subcutaneous mycotic lesions. In this case report, we present the case of a 47-year-old female patient who presented with swelling on the lateral aspect of the left lower leg above the lateral malleolus. Aspirate from the site was sent for histopathological examination for detection of the fungus. After microbiological investigation, a rare fungus isolated in this patient was identified as *Aquastroma magniostiolata *by carrying out sequencing at a reference center.

## Introduction

Phaeohyphomycosis is a disease caused by molds with melanin pigments in the cell wall. Phaeohyphomycosis is diagnosed by the appearance of pigmented hyphae or yeast cells in tissue [1]. More than 130 fungal species belonging to 70 diverse genera have been reported as causative agents for human and animal phaeohyphomycosis [2]. Commonly reported agents include *Exophiala jeanselmei*, *Wangiella dermatitis*, *Cladophiala bantiana*, *Scedosporium prolificans*, *Alternaria*, *Aureobasidium*, *Bipolaris*, *Curvularia *[3].

In this case, we studied phaeohyphomycosis caused by a rare fungus. It was isolated and identified by carrying out sequencing at a reference center as *Aquastroma magniostiolata*. After a thorough literature search on the internet and in textbooks of mycology, there are no case reports or reported infections of *Aquastroma magniostiolata* to date. Hence, this case report will lead the path for further clinical features of this disease and guidance for the management of such cases.

## Case presentation

A 47-year-old woman, working as a house-help, presented to the surgery outpatient department (OPD) with a complaint of localized swelling over the lateral aspect of left her lower leg above the lateral malleolus for eight months.

**Figure 1:**
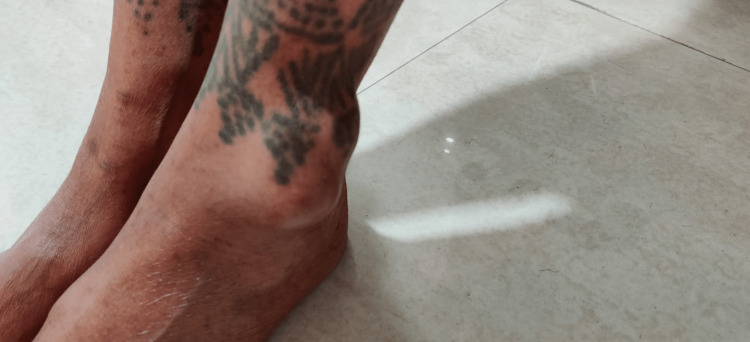
Nodular lesion on the lateral aspect of the lower leg.

The patient was apparently healthy one year ago when she noticed a small-sized (gram grain) nodular lesion on the lateral aspect of her lower leg above the lateral malleolus. It was a painless, soft, fixed, non-adherent, non-tender swelling. She did not have any inflammatory features, abnormal sensation, or skin pigmentation over the affected area. She was not experiencing any difficulty in walking or movement of lower limb but she had noticed the slight lateral deviation of the left feet and an increase in the left first toe web space after four months (Figure 1). The swelling gradually increased in size over four months and became cystic in consistency but remained painless. She gradually developed pain near the affected area and difficulty in walking after approximately eight months of onset of swelling in the affected limb.

There was no history of any pus or granular discharge, abscess formation, bony involvement, or fracture or similar lesions at any other body site. Any history of trauma at the affected part/limb could not be recalled by the patient. The patient was seronegative for human immunodeficiency virus 1 and 2 and hepatitis B surface antigen. Complete blood count, renal function test, and liver function test were within normal limits. There was no history of any chronic illness, diabetes mellitus, and steroid intake.

The patient was an average-built female with normal general and systemic examination. On local examination, single, irregularly shaped, soft multi-locular cystic, non-tender, mobile swelling measuring 4.5 × 4 × 3 cm was present on the left leg 3-4 cm above the lateral malleolus. A scar of a treated lower limb surgery (below the knee) was present, and old tattoo marks were present at and around the affected area. Tattooing was done in childhood.

**Figure 2:**
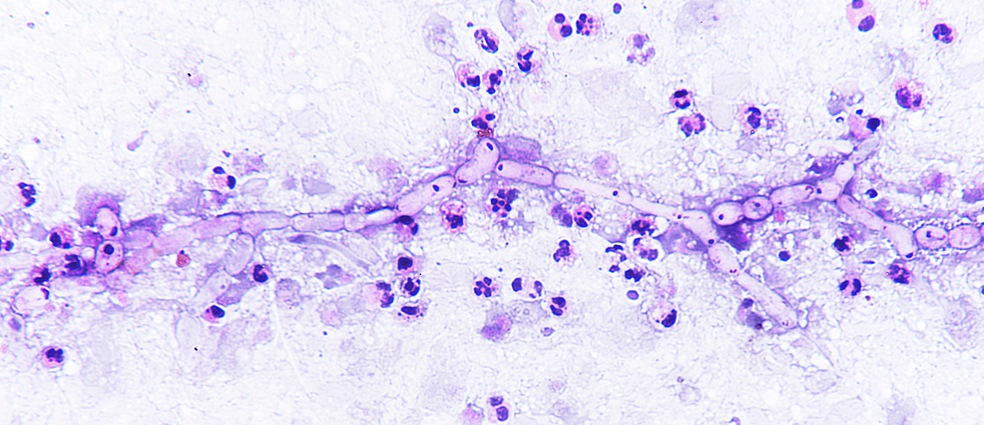
The presence of fungal hyphae with septations and acute angle branching with few fruiting bodies in a necrotic background with polymorphs and cellular debris.

The patient visited the surgery OPD twice during the entire duration. In the first visit, radiological and histopathology investigations were done. The ultrasonographic diagnosis was suggestive of subcutaneous septate cyst may be either pyogenic or infected sebaceous cyst. X-ray of the ankle joint showed the presence of subcutaneous soft tissue swelling without any bone involvement. In the same visit, fine-needle aspiration cytology was done in which 0.5 mL yellowish slightly thick fluid was aspirated. Cytology of the aspirate showed the presence of single and multiple groups of fungal hyphae with septations and acute angle branching with few fruiting bodies in a necrotic background with polymorphs and cellular debris (Figure 2). Fungal structures were highlighted in the periodic acid-Schiff stain. There were no granulomas. Zeihl-Neelsen stain for acid-fast bacilli was negative. A final impression of “subcutaneous fungal cyst-phaeohyphomycosis” was made and microbiological processing was advised. Antifungals were not administered during this visit.

**Figure 3:**
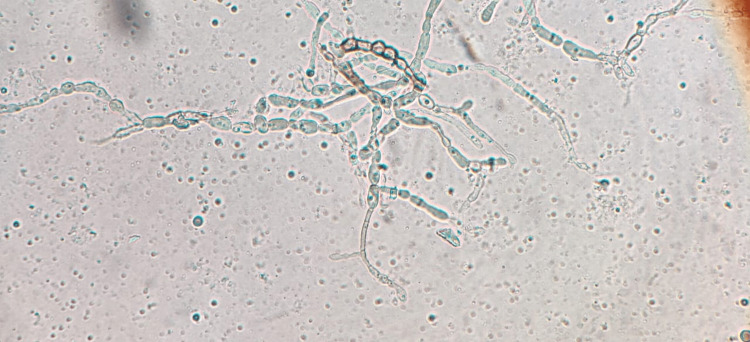
KOH mount of the aspirate showing the presence of irregularly swollen, septate, branched hyphae that were variably dark brown and hyaline spherical elements in short chains resembling chlamydospores.

**Figure 4:**
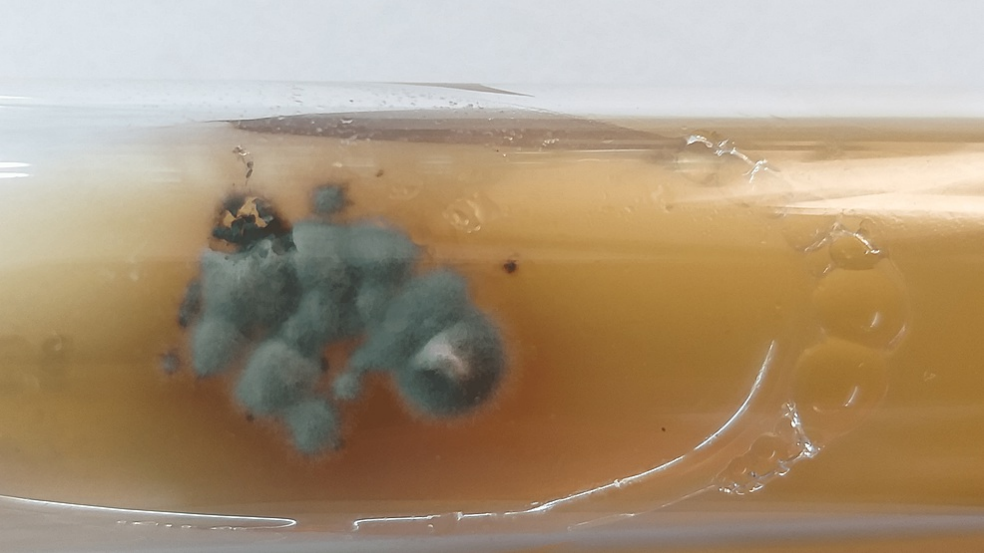
Growth on Sabouraud dextrose agar showing greenish velvety restricted colonies after two weeks of incubation.

After eight days in the second OPD visit, KOH mount and fungal culture were advised. 10% KOH preparation of the aspirate showed the presence of irregularly swollen, septate, branched hyphae that were variably dark brown and hyaline spherical elements in short chains resembling chlamydospores (Figure 3). On Calcofluor white stain 10% KOH preparation of aspirate, fungal elements were seen as fluorescent light blue-colored irregularly swollen branched hyphal structures. Fungal culture was inoculated on Sabouraud dextrose agar (SDA) slants, both with and without cycloheximide, and incubated at 25°C and 37°C. Growth was observed on the slants incubated at 25°C after one week as greenish velvety restricted colonies with a dark reverse that changed to black velvety growth in the next two weeks (Figure 4). Multiple lactophenol cotton blue (LPCB) preparations of the fungal growth at different incubation periods showed dematiaceous and hyaline branched hyphae with few septations without any conidiophores or conidia. Slide culture was inoculated but also yielded dematiaceous and hyaline sterile hyphae without any sporulating structures. The culture isolate was sent to the National Culture Collection of Pathogenic Fungi (NCCPF), Postgraduate Institute of Medical Education & Research (PGIMER), Chandigarh, for identification where it was identified as *Aquastroma magniostiolata* by DNA sequencing.

Though the patient was in occasional telephonic contact, she could not come for definitive treatment after the availability of the final identification due to the coronavirus disease 2019 pandemic. When contacted over the phone to inquire about her current condition, the patient informed that she had consulted locally for increasing discomfort and swelling of the legs and it was excised two months back, following which the symptoms were relieved.

## Discussion

Phaeohyphomycosis was first introduced by Ajello and colleagues [4]. It is an infection caused by fungi that are characterized by brown to black coloration of their vegetative or spore walls. These melanized fungi are present worldwide in soil, thorn, wood splinters, and decaying plant material. A common route of transmission is penetration injury with conidia containing soil or plant material [1]. Sharma et al. reviewed the literature on subcutaneous phaeohyphomycosis reported from India and predominantly observed involvement of the extremities, which are more prone to trauma [5]. As phaeohyphomycosis includes diverse pathogenic fungal agents, it is unlikely that there can be consistent unifying themes accounting for the virulence potential of all agents [3].

Phaeohyphomycosis is predominantly seen in males, and the reason might be due to more involvement in outdoor activities. However, in this case, the patient was a female who was involved in house-help work [1].

At times, the substance initiating the trauma can be recovered when pus is taken from lesions at the inoculation site; however, in this case, it could not be traced as the surgical management of the patient had to be taken locally due to the COVID-19-induced lockdown period.

The diagnosis of subcutaneous phaeohyphomycosis is difficult because of the clinical polymorphism of the lesions. Primarily, cystic lesions are seen in adults [1]. Cysts in immunocompetent patients are always chronic and rarely asymptomatic [1]. These can have little observable clinical changes. The diagnosis is usually established with histological examination and culture.

In tissue, hyphae may appear regular and uniform in diameter or may be irregular with many swollen cells. Some fungi may appear hyaline in tissue due to scant production of melanin, although they are melanized in culture. In this case, the darkly pigmented colonies were processed for sporulation as they could not be sporulated on available culture media, including water culture. After all efforts with the traditional methods of identification, it was suspected as an unusual dematiaceous fungus. Therefore, the culture was sent to NCCPF Chandigarh and it was identified as Aquastroma magniostiolata on DNA sequencing. A combination of microscopic examination, culture, and molecular approach proved appropriate in the final identification of the pathogen. As the infective etiology was not considered clinically, the specimen was not sent for microbiological culture initially; however, after the histopathogical report, the sample was sent for fungal culture and the rare fungus was identified.

Pai et al. reported a case of subcutaneous phaeohyphomycosis where the diagnosis was made based on histopathological examination as the fungus could not be revealed on KOH mount and culture but the reason was not mentioned [6].

Manoharan et al. reported a case of a subcutaneous pheomycological cyst on a patient’s left leg in which diagnosis was made based on the histopathology report as clinical suspicion was not of infective etiology and a sample was not sent for culture; hence, the exact species was not identified [7].

Aquastroma magniostiolata is a completely new fungus isolated from human clinical samples. After a search of online medical literature, we noted that it was not reported in human clinical samples. It is among the seven known genera in the family Parabambusicolaceae [7]. It is an aquatic saprobe reported from Japan [8] and isolated from a collection of dried stems of a dicotyledonous plant in Thailand [8]. The Parabambusicolaceae is created to accommodate *Aquastroma *and *Parabambusicola *genera nova, as well as two unnamed Monodictys species. The Parabambusicolaceae is characterized by depressed globose to hemispherical ascomata with or without surrounding stromatic tissue, and multi-septate, clavate to fusiform, hyaline ascospores which we could not detect as it could not sporulate [9].

Various dematiaceous fungi have been reported from phaeohyphomycotic cysts. Various unusual fungal agents such as *Phialemoniopsis ocularis* [10], *Phaeoacremonium inflatipes* [11], *Exophiala oligosperma* [12], *Exophiala spinifera *[13], *Medicopsis romeroi* (*Pyrenochaeta romeroi*) [14,15] are the reported from of subcutaneous phaeohyphomycosis.

Chhonkar et al. [16] reported cases of cutaneous phaeohyphomycosis based on histopathology, KOH, and culture on SDA in which the presence of dematiaceous fungi was revealed but a specific fungus was not mentioned.

Most of the time, the diagnosis of phaeohyphomycosis is made based on histopathological examination, and patients are managed without knowing the specific causative fungi [5,6,17]. It remains undetected due to a lack of awareness and culture facilities, unavailability of molecular diagnostic services, and unfamiliar morphological features. Hence, the exact distribution and prevalence of the existing known fungal agents or newer and rare fungi causing phaeohyphomycosis remained mysterious. Correct identification of genus and species may help to establish the clinical significance of the isolate, predict the extent of infection, and prognosis. It may help to standardize the susceptibility pattern of antifungal agents.

Regarding management, the local infection may be treated with excision or cryotherapy without knowing the exact etiology. Dematiaceous fungi are most susceptible to voriconazole, itraconazole and posaconazole, whereas ketoconazole and fluconazole have limited activity. Amphotericin-B and 5-flucytosine may also be used as treatment modalities. Systemic disease is often refractory to therapy. Successful treatment of these lesions includes complete excision [1]. In this study, though the patient had to excise it locally, telephonic follow-up is still ongoing to monitor the re-occurrence of the infection.

## Conclusions

There is an extensive list of fungi that are present in the environment causing phaeohyphomycosis. *Aquastroma magniostiolata *is also one of the new saprophytes isolated from subcutaneous cystic lesions which have not been reported from human clinical samples to date. The identification of the agent is important to know the epidemiology of the etiological agent. Reporting rare cases is also crucial to help the management of future cases occurring due to the same fungi. Microscopy, culture, and sequencing proved critical in identifying the particular fungal agent of phaeohyphomycosis.
